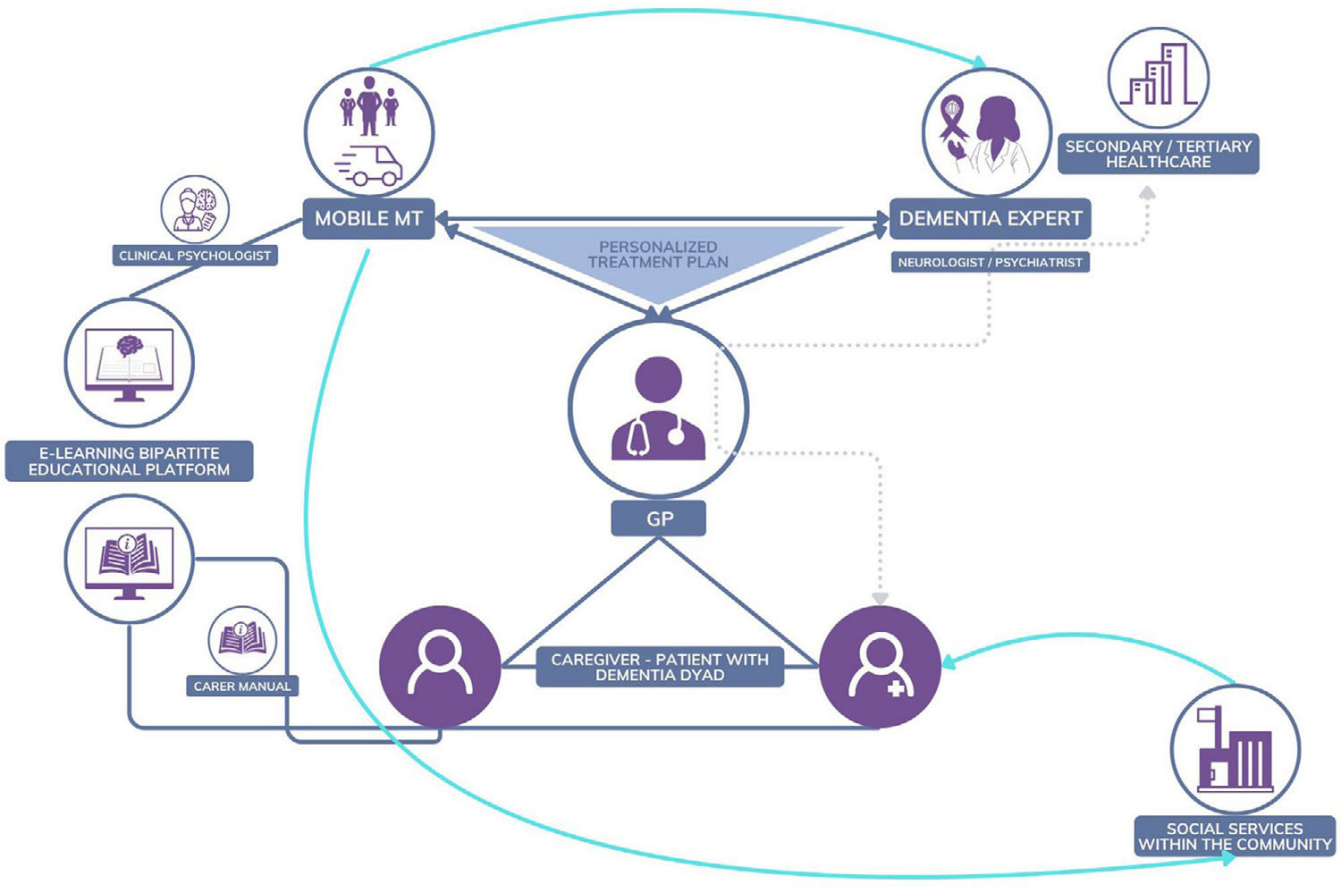# North Macedoniainterprofessional dementia care (NOMAD) – personalized careplans for people with dementia and caregiver psychoeducation delivered at home by interprofessional teams

**DOI:** 10.1002/alz.095474

**Published:** 2025-01-09

**Authors:** Gabriela Novotni, Marija Taneska, Antoni Novotni, Julia Fischer, Svetlana Iloski, Andrea Ivanovska, Vesna Dimitrova, Ljubisha Novotni, Milos Milutinovic, Boban Joksimoski, Ivan Chorbev, Shpresa Hasani, Vildan Dogan, Timo Grimmer, Alexander Kurz

**Affiliations:** ^1^ University Clinic of Neurology, Skopje, n.a. Macedonia, The former Yugoslav Republic of; ^2^ Institute for Alzheimer’s Disease and Neuroscience, Skopje, n.a. Macedonia, The former Yugoslav Republic of; ^3^ University Clinic of Psychiatry, Skopje, n.a. Macedonia, The former Yugoslav Republic of; ^4^ Technical University of Munich, School of Medicine and Health, Munich, Bavaria Germany; ^5^ Institute for Alzheimer's Disease and Neuroscience, Skopje, n.a. Macedonia, The former Yugoslav Republic of; ^6^ Faculty of Computer Science and Engineering, Ss. Cyril and Methodius University, Skopje, n.a. Macedonia, The former Yugoslav Republic of; ^7^ Center for Cognitive Disorders, Technical University of Munich, School of Medicine and Health, Klinikum rechts der Isar, Munich, Bavaria Germany; ^8^ Technical University of Munich, School of Medicine and Health, Klinikum rechts der Isar, Munich, Bavaria Germany

## Abstract

**Background:**

The increasing number of people living with dementia and its burden on families and systems particularly in low‐ andmiddle‐income countries require comprehensive and ecient post‐diagnostic management. This study aimed to explore the acceptability and ecacy of a multi‐professional case management and psychoeducation model (North Macedonia Interprofessional Dementia Care, or NOMAD) delivered bymobile teams for people with dementia and their caregivers in North Macedonia.

**Method:**

We conducted a two‐arm randomized controlled trial comparing the intervention with treatment as usual. Participants were recruited from 12 general practitioner (GP) oces in the Skopje region. The NOMAD intervention included the delivery of a personalized care plan over four home visits to dyads of people with dementia and their caregivers by a team including a dementia nurse and a social worker, in collaboration with GPs and dementia experts, and the introduction of a caregiver manual. We assessed caregivers’ depressive symptoms, burden, and quality of life and the neuropsychiatric symptoms, daily living activities, and service utilization of people with dementia at baseline and follow‐up; we also assessed the acceptability of the intervention by analyzing case notes and attendance rates.

**Result:**

One hundred and twenty dyads were recruited and randomized to either the control (n = 60) or the intervention group (n = 60). At follow‐up, caregivers in the intervention group had, on average, scores that were 2.69 lower for depressive symptoms (p = 0.012), and people with dementia had, on average, 11.32 fewer neuropsychiatric symptoms (p = 0.009) and used, on average, 1.81 fewer healthcare services (p < 0.001) compared to the control group. The completion of the home visits was 100%, but the intervention’s acceptability was underpinned by relationship building, GP competencies, and resources to support families with dementia. NOMAD is the first casemanagement, non‐pharmacological, and multi‐professional intervention tested in North Macedonia.

**Conclusion:**

The trial showed that it is effective in reducing caregivers’ depressive symptoms and neuropsychiatric symptoms in people with dementia and the burden on health and social care services, and it is acceptable for families. Implementing NOMAD in practice will require building primary care capacity and recognizing dementia as a national priority.